# Temporal trends in the incidence and outcomes of shock-refractory ventricular fibrillation out-of-hospital cardiac arrest

**DOI:** 10.1016/j.resplu.2024.100597

**Published:** 2024-03-08

**Authors:** Abdulrahman Alhenaki, Zainab Alqudah, Brett Williams, Emily Nehme, Ziad Nehme

**Affiliations:** a Department of Paramedicine, Monash University, Frankston, Victoria, Australia; b Prince Sultan ibn Abdulaziz College for Emergency Medical Services, King Saud University, Riyadh, Saudi Arabia; c Faculty of Allied Medical Sciences, Jordan University of Science and Technology, Irbid, Jordan; d Centre for Research and Evaluation, Ambulance Victoria, Blackburn North, Victoria, Australia; e School of Public Health and Preventive Medicine, Monash University, St Kilda., Victoria, Australia

**Keywords:** Out-of-hospital cardiac arrest, Ventricular fibrillation, Emergency medical services, Cardiopulmonary resuscitation

## Abstract

**Aim:**

We aimed to describe trends in the incidence and outcomes of refractory ventricular fibrillation (RVF) compared to non-refractory ventricular fibrillation (non-RVF) in out-of-hospital cardiac arrest (OHCA).

**Methods:**

Between 2010 and 2019, we included all OHCA cases involving adults ≥ 16 years old with an initial shockable rhythm and who received an attempted resuscitation by Emergency Medical Services (EMS) or a bystander shock prior to EMS arrival in Victoria, Australia. Trends in incidence and survival outcomes over the study period were examined. Adjusted logistic regression analyses were conducted to examine factors associated with RVF, as well as the association of RVF on survival to hospital discharge. RVF refers to patients receiving three or more consecutive shocks without a return of spontaneous circulation (ROSC).

**Results:**

Of the 57,749 OHCA attended by EMS, 7,267 met the inclusion criteria. Of these, 4,168 (57.4%) were non-RVF and 3,099 (42.6%) were RVF. The incidence of RVF decreased significantly from 7.7 per 100,000 population in 2010 to 5.6 per 100,000 population in 2019 (*p*-trend = 0.01). Survival to hospital discharge increased significantly for both the RVF and non-RVF groups (26% vs 41% in 2010 to 31% vs 53% in 2019, *p*-trend = 0.004 for RVF; and *p*-trend = 0.01 for non-RVF). Compared to non-RVF, RVF was associated with reduced odds of survival to hospital discharge (Odds Ratio = 0.503 [95% confidence interval 0.448 – 0.565]). Factors associated with a lower likelihood of RVF and improved survival to hospital discharge included being witnessed to arrest by EMS, receiving bystander defibrillation and bystander cardiopulmonary resuscitation (CPR).

**Conclusion:**

The incidence of RVF is declining, and survival rates are improving. Early treatment of VF patients with bystander CPR and defibrillation is likely to reduce RVF incidence.

## Introduction

Globally, out-of-hospital cardiac arrest (OHCA) affects 55 adults per 100,000 people, with a survival rate of 8.8%.^1^, ^2^ Approximately one-quarter of all EMS-treated OHCA events present with Ventricular Fibrillation (VF) or pulseless Ventricular Tachycardia (VT) as the initial cardiac rhythm,^1^, ^3^ and these patients are reported to have better survival outcomes, between 10% and 30%.^1^ However, despite receiving multiple defibrillations, repeated doses of epinephrine and anti-arrhythmic drugs, more than half of patients with VF/VT do not respond to conventional treatment and often do not survive.^4^ This condition is known as shock-refractory Ventricular Fibrillation (RVF).

Although the definition of RVF is still unclear,^5^ it is often defined as patients with at least three consecutive defibrillation attempts without achieving (ROSC).^6^, ^7^ Many studies exploring RVF included a combination of refractory and recurrent VF cases, where recurrent VF refers to the transient conversion of ventricular arrythmias followed by their rapid recurrence, usually between rhythm analysis cycles.^7^ Challenges in obtaining and analysing electrocardiogram records real-time make distinguishing between the two groups impractical.^7^

While changes in the incidence of shockable rhythms over time have been reported in the literature,^8^ changes in the incidence of RVF and non-RVF remain under investigated. To our knowledge, only one study, conducted in Japan, reported the long-term incidence and outcomes of RVF. The incidence of RVF was 0.5 per 100,000 population per year, and this remained unchanged over the study period.^8^ On the other hand, studies have reported a high survival to hospital discharge rate among RVF patients undergoing extracorporeal membrane oxygenation (ECMO) treatment.^9^ However, its implementation poses challenges due to feasibility and resource demands.^10^ Furthermore, it is unclear if patient outcomes are changing over time for RVF, or whether its occurrence is potentially modifiable through community or EMS care provision. As such, there is a need to examine temporal patterns in the incidence and outcome of RVF relative to non-refractory cases of VF/VT.

In this study, we sought to provide an epidemiological analysis of the temporal trends in the incidence of RVF and non-RVF OHCA in Victoria, Australia. In addition, we aimed to identify risk factors for the development of RVF, and examine the impact of RVF on survival to hospital discharge.

## Methods

### Study design

A retrospective cohort study was performed using data from the Victorian Ambulance Cardiac Arrest Registry (VACAR). We included all adults aged ≥16 years old with an initial shockable rhythm and who received an attempted resuscitation by emergency medical services (EMS) or a bystander shock prior to EMS arrival between January 2010 and December 2019. We excluded cases of traumatic aetiology and cases where the number of administered shocks was not documented. Ethical approval for this study was obtained from the Monash University Human Research and Ethics Committee (Project ID: 36838).

### Setting

The State of Victoria, Australia, has a population of more than 6.6 million people, distributed across an area of 227,500 square kilometres. The EMS is operated by Ambulance Victoria, a single state-wide EMS system. Annually, Ambulance Victoria attends to more than 7,000 OHCA cases.^11^ The EMS has three levels of response to suspected cardiac arrest events, including Basic Life Support first responders, Advanced Life Support paramedics and intensive care paramedics. Local treatment guidelines follow the recommendations of the Australian and New Zealand Committee on Resuscitation (https://www.anzcor.org). First responders include firefighters and community volunteers who are trained in basic life support and Automated External Defibrillation (AED) administration. Advanced Life Support paramedics are authorised to perform defibrillation, administer intravenous adrenaline and fluid, and insert a supraglottic airway. Intensive care ambulance paramedics are authorised to conduct additional treatments and interventions, such as endotracheal intubation (including rapid sequence intubation), as well as administer a wider range of intravenous medications.

### Data source and definitions

For this study, we collected data from the VACAR. The registry was established in 1999, recording all OHCA cases attended by EMS in the state of Victoria. More than 150 data elements are collected by VACAR, including the Utstein-style descriptors^12^ and patient discharge outcomes from hospital records for cases that are transported to hospitals.^13^ In this study, RVF refers to all initially shockable arrests who received three or more consecutive defibrillations, either from EMS or bystander, without achieving ROSC. Non-RVF refers to all other initially shockable arrests, including those with less than three consecutive defibrillations, either from EMS or bystander, or non-consecutive defibrillation attempts. EMS-attempted resuscitation refers to any attempt at defibrillation or chest compressions by EMS.^12^ Resuscitation time refers to the total time from the commencement of EMS CPR to the end of CPR either upon achieving ROSC or upon terminating resuscitation efforts.

### Study outcomes

The primary outcome of this research was survival to hospital discharge. The secondary outcomes included the incidence of RVF and non-RVF and the number of patients who achieved ROSC and survived the event (pulse at hospital arrival).

### Data analysis

Statistical analyses were performed using STATA statistical software 18 (StataCorp, 2015, College Station, TX). All hypothesis tests were two-sided and p-values of less than 0.05 were considered statistically significant.

Arrest characteristics and survival outcomes are reported using descriptive statistics stratified by RVF and non-RVF status. To measure differences in baseline characteristics and survival outcomes, the chi-square test and Mann-Whitney U test were used, as appropriate. Long-term trends in survival to hospital discharge for RVF and non-RVF patients were assessed using a non-parametric test for trend.

Annual crude incidence rates of RVF and non-RVF were calculated per 100,000 population. The estimated residential population was obtained from the Australian Bureau of Statistics (https://www.abs.gov.au). Trends in incidence were assessed for the RVF and non-RVF groups using a non-parametric test for trend.^14^

Multivariable logistic regression models were used to identify: 1) factors associated with the development of RVF; and, 2) the association between RVF and survival to hospital discharge. Models were adjusted for: age in years, male sex, comorbidities, the presence of bystander cardiopulmonary resuscitation (CPR), whether the event was witnessed, defibrillation by a bystander, arrest aetiology, public location, metropolitan region, and year of cardiac arrest. To account for resuscitation time bias in the survival to hospital discharge model, we also performed a sensitivity analysis which included the total resuscitation time. Results from these models were reported as adjusted odds ratios (OR) with 95% confidence intervals (CI).

## Results

### Sample population

**Fig. 1:**
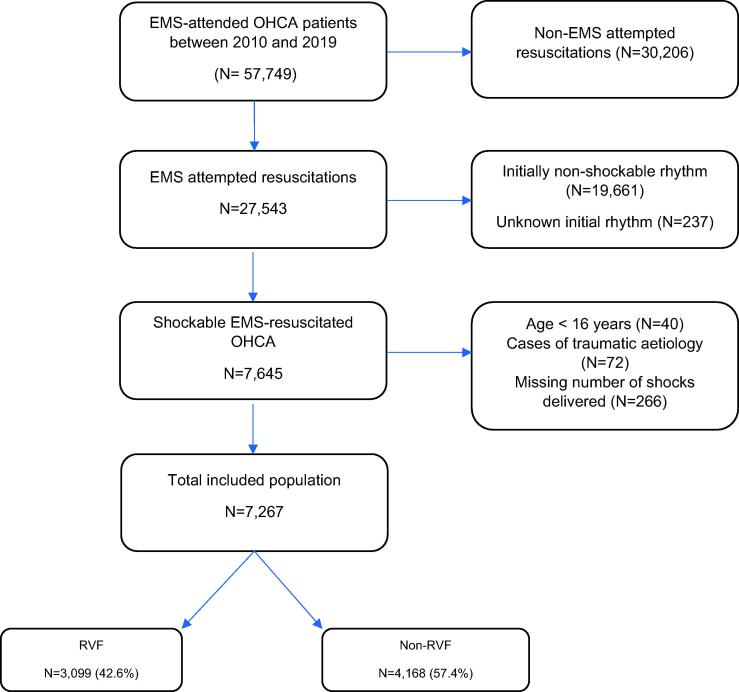
Selection of population between January 2010 and December 2019 in Victoria, Australia.

A total of 57,749 EMS-attended OHCA patients were captured by the registry between 2010 and 2019, of whom 7,267 (12.6%) involved adult, non-traumatic, initially shockable OHCA with an EMS attempted resuscitation or initial bystander shock. Of these, 4,168 (57.4%) and 3,099 (42.6%) were non-RVF and RVF, respectively (Fig. 1).

### Baseline characteristics and survival outcomes across groups

Table 1 shows baseline characteristics for RVF and non-RVF patients. Compared to non-RVF patients, RVF cases were less often witnessed by EMS (24.7% vs. 5.9%), shocked first by a bystander (11.0% vs. 6.6%) and received bystander CPR (83.5% vs. 79.5%) (P-value for all comparisons < 0.001). On the other hand, compared to the non-RVF group, the RVF group were more likely to be male (77.4% vs. 81.2%, *p* < 0.001), have presumed cardiac aetiology (95.2% vs. 96.9%, *p* < 0.001), be witnessed to arrest by a bystander (58.7% vs. 73.0%, *p* < 0.001) or not witnessed to arrest (16.5% vs. 21.5%, *p* < 0.001), shocked first by paramedics (82.2% vs. 84.5%, *p* = 0.008) or first responders (6.9% vs. 8.9%, *p* = 0.001) and arrest in private location (53.3% vs. 59.7%, *p* < 0.001). In addition, the median resuscitation time was shorter in the non-RVF group compared to the RVF group (20.5 vs 37.0 minutes, *p* < 0.001). The RVF group more often had hypertension, peripheral vascular disease and cardiac disease as comorbidities.

**Table 1:** Baseline characteristics.

	Overall N = 7,267	Non-refractory VF N = 4,168	Refractory VF N = 3,099	*p*-value	Missing
Age in years, median (IQR)	65 (54 – 76)	65 (54 – 76)	66 (55 – 76)	0.137	1
Male, n (%)	5,740 (79.0%)	3,225 (77.4%)	2,515 (81.2%)	<0.001	0
Comorbidity, n (%)					
No medical history	2,197 (30.2%)	1,295 (31.1%)	902 (29.1%)	0.071	
Cancer	359 (4.9%)	211 (5.1%)	148 (4.8%)	0.577	
Stroke	287 (3.9%)	156 (3.7%)	131 (4.2%)	0.294	
Hypertension	2,032 (28.0%)	1,121 (26.9%)	911 (29.4%)	0.019	
Hyperlipidaemia	1,201 (16.5%)	661 (15.9%)	540 (17.4%)	0.075	
Diabetes	912 (12.6%)	532 (12.8)	380 (12.3%)	0.523	
Heart Failure	551 (7.6%)	297 (7.1%)	254 (8.2%)	0.088	
Peripheral Vascular Disease	997 (13.7%)	524 (12.6%)	473 (15.3%)	0.001	
Renal Failure	190 (2.6%)	125 (3.0%)	65 (2.1%)	0.017	
COPD	329 (4.5%)	184 (4.4%)	145 (4.7%)	0.592	
Asthma	292 (4.0%)	171 (4.1%)	121 (3.9%)	0.670	
Substance Abuse	165 (2.3%)	96 (2.3%)	69 (2.2%)	0.828	
Neurodegenerative Disorder	54 (0.7%)	32 (0.8%)	22 (0.7%)	0.776	
Mental Illness	476 (6.6%)	315 (7.6%)	161 (5.2%)	<0.001	
Arrhythmia	561 (7.7%)	328 (7.9%)	233 (7.5%)	0.579	
Cardiac disease	1,513 (20.8%)	833 (20.0%)	680 (21.9%)	0.042	
Other conditions	424 (5.8%)	260 (6.2%)	164 (5.3%)	0.089	
Unknown conditions	579 (8.0%)	301 (7.2%)	278 (9.0%)	0.006	
Presumed cardiac aetiology, n (%)	6,973 (96%)	3,969 (95.2%)	3,004 (96.9%)	<0.001	0
Witness status, n (%)					32 (0.4%)
Witnessed by bystander	4,690 (64.8%)	2,437 (58.7%)	2,253 (73.0%)	<0.001	
Witnessed by EMS	1,195 (16.5%)	1,026 (24.7%)	169 (5.9%)	<0.001	
Not witnessed	1,350 (18.7%)	686 (16.5%)	664 (21.5%)	<0.001	
First shock provider, n (%)					68 (0.9%)
Paramedic	5,987 (83.2%)	3,368 (82.2%)	2,619 (84.5%)	0.008	0
First responder	557 (7.7%)	281 (6.9%)	276 (8.9%)	0.001	0
Bystander*	655 (9.1%)	451 (11.0%)	204 (6.6%)	<0.001	0
Bystander CPR, n (%) *	4,927 (81.6%)	2,609 (83.5%)	2,318 (79.5%)	<0.001	0
Arrest location, n (%)					
Private location	4,072 (56%)	2,221 (53.3%)	1,851 (59.7%)	<0.001	0
Aged care	239 (3.3%)	154 (3.7%)	85 (2.7%)	0.024	
Public location	2,266 (31.1%)	1,278 (30.7%)	988 (31.9%)	0.267	0
Other	690 (9.5%)	515 (12.4%)	175 (5.7%)	<0.001	
Metropolitan region, n (%)	5,083 (70.0%)	2,884 (69.2%)	2,199 (70.7%)	0.105	
EMS response time, median (IQR)*	7.9 (6.1 – 10.5)	7.8 (6.1 – 10.5)	7.9 (6.1 – 10.4)	0.943	0
Resuscitation time, median (IQR)	31 (10 – 46)	20.5 (5 – 42)	37 (23 – 51)	<0.001	50 (0.7%)
Scene outcomes, n (%)					
Died at scene or transit	2,669 (36.7%)	1,177 (28.3%)	1,492 (48.1%)	<0.001	
Transported with CPR	410 (5.6%)	177 (4.3%)	233 (7.5%)	<0.001	
Transported with ROSC	4,187 (57.6%)	2,813 (67.5%)	1,374 (44.3%)	<0.001	
Survival outcomes, n (%)					
Pre-hospital ROSC	4,733 (65.1%)	3,111 (74.6%)	1,622 (52.3%)	<0.001	0
Event survival	4,175 (57.5%)	2,806 (67.4%)	1,369 (44.2%)	<0.001	4 (0.1%)
Discharged alive	2,695 (37.1%)	1,902 (45.6%)	793 (25.6%)	<0.001	79 (1.1%)

*Exclude Ems-witnessed cases.

IQR stands for interquartile range; EMS, Emergency Medical Services; CPR, Cardiopulmonary Resuscitation; ROSC, Return of Spontaneous Circulation; COPD, Chronic Obstructive Pulmonary Disease..

Survival outcomes revealed substantial difference between non-RVF and RVF cases. Compared to non-RVF cases, RVF cases had lower rates of pre-hospital ROSC (74.6% vs 52.3%, *p* < 0.001), event survival (67.4% vs 44.2%, *p* < 0.001) and survival to hospital discharge (45.6% vs. 25.6%, *p* < 0.001).

### Trends in incidence and survival

**Fig. 2:**
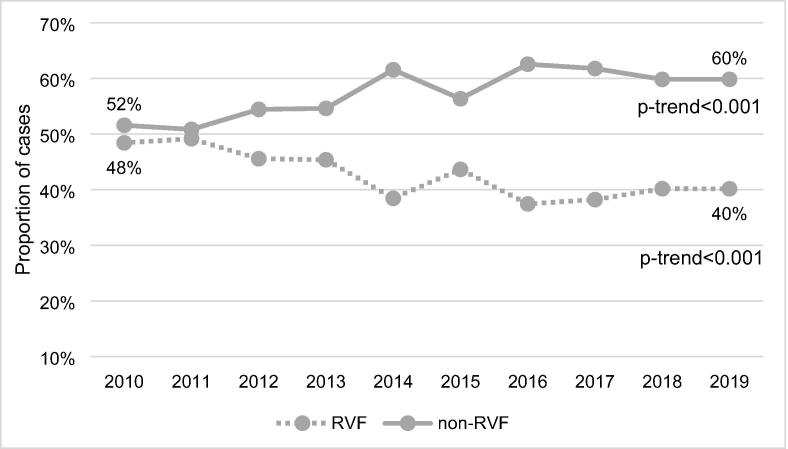
Unadjusted trends in RVF and non-RVF cases over the study period.

**Fig. 3:**
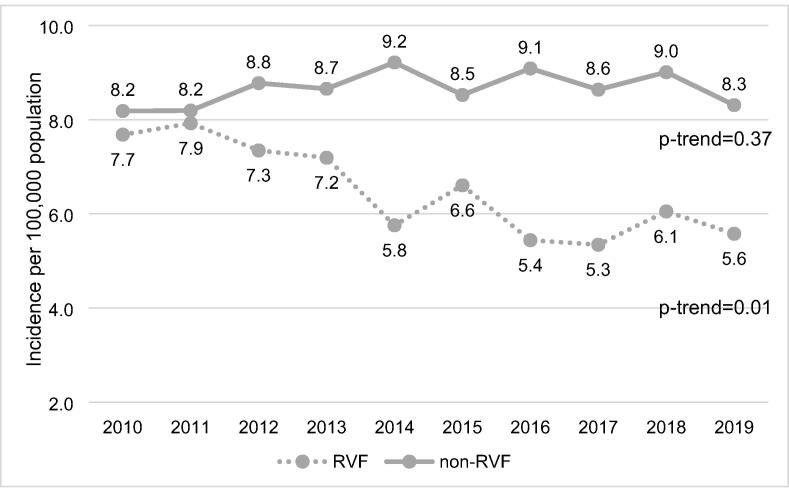
Crude incidence of RVF and non-RVF cases over the study period.

Trends in the proportion of RVF and non-RVF cases over the study period are presented in Fig. 2. The proportion of non-RVF cases increased from 52% in 2010 to 60% in 2019 (*p*-trend < 0.001). In contrast, the proportion of RVF cases decreased from 48% in 2010 to 40% in 2019 (*p*-trend < 0.001). Trends in incidence of RVF and non-RVF cases over the study period are shown in Fig. 3. The overall incidence of RVF cases decreased significantly from 7.7 cases per 100,000 population in 2010 to 5.6 cases per 100,000 population in 2019 (*p*-trend = 0.01). In comparison, there was no significant change in the incidence of non-RVF cases (*p*-trend = 0.37).

**Fig. 4:**
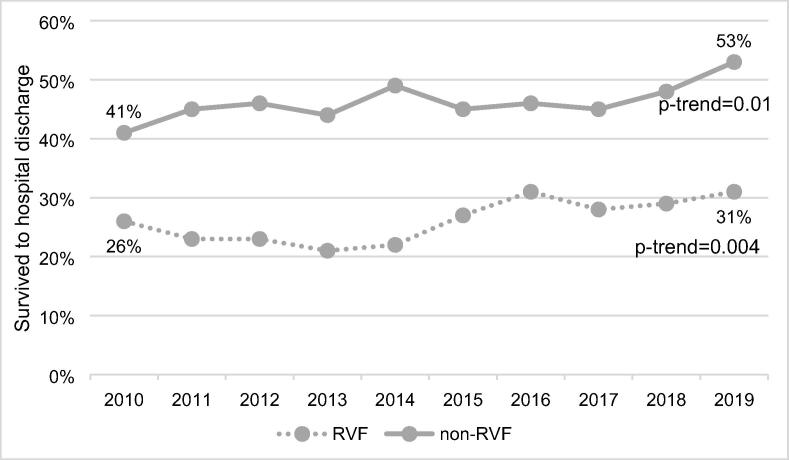
Unadjusted trends in survival to hospital discharge for RVF and non-RVF cases over the study period.

Fig. 4 shows the unadjusted trend in survival to hospital discharge in RVF and non-RVF patients over the study period. Survival to hospital discharge in the non-RVF cohort increased significantly from 41% in 2010 to 53% in 2019 (*p*-trend = 0.01). Similarly, the proportion of RVF patients who survived to hospital discharge increased significantly from 26% in 2010 to 31% in 2019 (*p*-trend = 0.004).

### Factors associated with RVF

Table 2 shows the factors associated with the development of RVF. Male sex (OR 1.186, 95% CI:1.047, 1.377), presumed cardiac aetiology (OR 1.446, 95% CI: 1.102, 1.898) and hypertension (OR 1.165, 95% CI: 1.018, 1.334) were associated with increased odds of RVF. The following variables were associated with decreased odds of RVF: being witnessed to arrest by EMS (OR 0.148, 95% CI: 0.119, 0.185), initially shocked by bystander (OR 0.495, 95% CI: 0.412, 0.594), bystander CPR (OR 0.860, 95% CI: 0.750, 0.985), diabetes (OR 0.839, 95% CI: 0.717, 0.983), renal failure (OR 0.677, 95% CI: 0.487, 0.941) and mental illness (OR 0.674, 95% CI: 0.544, 0.835). The odds of RVF decreased by 4% for every year of the study period (OR 0.963, 95% CI: 0.947, 0.980).

**Table 2:** Multivariable analyses.

	Risk-adjusted odds of developing RVF		Risk-adjusted odds of survival to hospital discharge		Risk-adjusted odds of survival to hospital discharge, including resuscitation time	
	OR (95% CI)	P-value	OR (95% CI)	P-value	OR (95% CI)	P-value
Age, per year increase	1.001 (0.998 – 1.005)	0.528	0.968 (0.964 – 0.972)	<0.001	0.965 (0.961 – 0.969)	<0.001
Gender						
Female	Reference		Reference		Reference	
Male	1.186 (1.047 – 1.377)	0.007	1.008 (0.876 – 1.159)	0.916	1.017 (0.882 – 1.174)	0.813
Pre-existing conditions						
No conditions	Reference		Reference		Reference	
Cancer	0.964 (0.764 – 1.217)	0.759	0.643 (0.483 – 0.875)	0.003	0.614 (0.459 – 0.822)	0.001
Stroke	1.082 (0.838 – 1.396)	0.546	0.745 (0.541 – 1.026)	0.071	0.700 (0.505 – 0.969)	0.031
Hypertension	1.165 (1.018 – 1.334)	0.026	0.980 (0.841 – 1.142)	0.799	0.962 (0.823 – 1.125)	0.626
Hyperlipidaemia	1.034 (0.882 – 1.212)	0.682	1.178 (0.981 – 1.415)	0.079	1.189 (0.986 – 1.434)	0.071
Diabetes	0.839 (0.717 – 0.983)	0.030	0.730 (0.607 – 0.878)	0.001	0.717 (0.593 – 0.865)	0.001
Heart Failure	1.115 (0.908 – 1.370)	0.298	0.528 (0.398 – 0.701)	<0.001	0.535 (0.402 – 0.712)	<0.001
Peripheral Vascular Disease	1.148 (0.981 – 1.345)	0.086	1.161 (0.964 – 1.399)	0.116	1.143 (0.945 – 1.381)	0.169
Renal Failure	0.677 (0.487 – 0.941)	0.020	0.422 (0.270 – 0.659)	<0.001	0.423 (0.270 – 0.662)	<0.001
COPD	1.118 (0.873 – 1.431)	0.379	0.661 (0.478 – 0.913)	0.012	0.633 (0.455 – 0.882)	0.007
Asthma	0.988 (0.764 – 1.278)	0.926	1.042 (0.778 – 1.395)	0.784	1.030 (0.763 – 1.390)	0.848
Substance Abuse	1.117 (0.794 – 1.571)	0.525	0.800 (0.545 – 1.173)	0.253	0.784 (0.529 – 1.162)	0.225
Neurodegenerative Disorder	1.072 (0.589 – 1.948)	0.821	0.370 (0.137 – 0.999)	0.050	0.380 (0.143 – 1.012)	0.053
Mental Illness	0.674 (0.544 – 0.835)	<0.001	0.914 (0.718 – 1.164)	0.467	0.848 (0.664 – 1.085)	0.189
Arrhythmia	0.896 (0.738 – 1.088)	0.270	1.354 (1.080 – 1.697)	0.009	1.351 (1.072 – 1.702)	0.011
Cardiac disease	1.001 (0.869 – 1.152)	0.992	0.913 (0.775 – 1.076)	0.275	0.904 (0.765 – 1.068)	0.235
Other conditions	0.961 (0.772 – 1.195)	0.718	0.838 (0.663 – 1.058)	0.137	0.797 (0.627 – 1.014)	0.065
Unknown conditions	1.176 (0.975 – 1.419)	0.090	0.729 (0.592 – 0.896)	0.003	0.715 (0.578 – 0.884)	0.002
Presumed cardiac	1.446 (1.102 – 1.898)	0.008	3.434 (2.464 – 4.786)	<0.001	3.604 (2.581 – 5.033)	<0.001
Witness status						
Unwitnessed	Reference		Reference		Reference	
Witnessed by bystander	0.996 (0.879 – 1.128)	0.947	1.881 (1.604 – 2.207)	<0.001	1.882 (1.600 – 2.213)	<0.001
Witnessed by EMS	0.148 (0.119 – 0.185)	<0.001	13.547 (10.577 – 17.350)	<0.001	12.161 (9.447 – 15.655)	<0.001
Shocked by bystander	0.495 (0.412 – 0.594)	<0.001	1.655 (1.368 – 2.001)	<0.001	1.491 (1.223 – 1.817)	<0.001
Bystander CPR	0.860 (0.750 – 0.985)	0.030	1.422 (1.193 – 1.694)	<0.001	1.462 (1.224 – 1.747)	<0.001
Public location	0.889 (0.790 – 1.002)	0.054	1.950 (1.711 – 2.223)	<0.001	1.867 (1.633 – 2.134)	<0.001
Metropolitan region	1.037 (0.931 – 1.156)	0.505	1.574 (1.392 – 1.780)	<0.001	1.566 (1.381 – 1.775)	<0.001
Year, per year increase	0.963 (0.947 – 0.980)	<0.001	1.035 (1.015 – 1.055)	0.001	0.970 (0.949 – 0.991)	0.006
RVF			0.503 (0.448 – 0.565)	<0.001	0.585 (0.519 – 0.659)	<0.001
Resuscitation time					0.983 (0.980 – 0.985)	<0.001

OR stands for Odds Ratio; CI, Confidence Interval; EMS, Emergency Medical Services; CPR, Cardiopulmonary Resuscitation; COPD, Chronic Obstructive Pulmonary Disease; RVF, Refractory Ventricular Fibrillation.

### Impact of RVF on survival

Table 2 shows the association between RVF and survival to hospital discharge after adjustment for arrest factors. RVF was associated with a 50% reduction in the odds of survival to hospital discharge (OR 0.503, 95% CI: 0.448, 0.565). The odds of survival increased by 3.5% annually (OR 1.035, 95% CI: 1.015, 1.055).

To determine whether our results were influenced by resuscitation time, we repeated the above analysis by including resuscitation time in the model (Table 2). In this model, increasing resuscitation time was significantly associated with reduced odds of survival to hospital discharge (OR 0.983, 95% CI: 0.980, 0.985), and only modestly attenuated the impact of RVF on survival to hospital discharge (OR 0.585, 95% CI: 0.519, 0.659).

## Discussion

Our analysis of EMS-attended OHCA over a 10-years period in Victoria, Australia suggests that the incidence of initially shockable arrests involving RVF is decreasing significantly year on year. Our results also suggest that RVF is associated with a 50% reduction in the odds of survival, even after adjusting for confounding factors, including resuscitation time. Importantly, the likelihood of survival to hospital discharge increased over time for both non-RVF and RVF cases. Our multivariable models suggest that EMS-witnessed arrests, initial defibrillation by a bystander and bystander CPR were associated with a lower likelihood of RVF and improved survival to hospital discharge. These findings suggest that the development of RVF may be mitigated by optimisations in the chain of survival and the delivery of early basic life support.

In this study, the long-term trend of RVF incidence reduced significantly over time from 7.7 to 5.6 cases per 100,000 population. A study conducted in Osaka prefecture in Japan between 1998 and 2006 which included 1733 patients with VF, reported different findings.^8^ They reported that the overall incidence of shock-resistant VF remained stable, despite improvements in their chain of survival. However, the definition of RVF differed from that used in our study. They defined RVF as VF/VT that persisted after hospital arrival and which was preceded by at least one EMS shock. This definition might have omitted patients who achieved ROSC after three or more shocks in the prehospital setting. This may partly explain their very low incidence of 0.5 cases per 100,000 population.

It is not clear why the incidence of RVF reduced over time in our study. However, our results suggest that bystander interventions were associated with reduced odds of RVF, and bystander administration of CPR and defibrillation have increased over time in our region.^11^ Data from Victoria, Australia, showed that, for OHCA events witnessed by bystanders who received an attempted resuscitation by EMS, the rate of bystander CPR increased from 70% to 77% between 2011–2012 and 2020–2021 (*p* = 0.01).^11^ Further, initial defibrillation by paramedics reduced from 81% to 77% (*p* < 0.001), and this was met by close to a doubling in the use of AEDs by bystanders (7% to 13%, *p* = 0.002) over the same period.^11^ Efforts to promote bystander-administered CPR and prompt defibrillation may further reduce the incidence of RVF.

RVF was associated with decreased odds of survival in our study. The prognosis of RVF is poor in the published literature.^7^, ^15^ However, a number of modifiable factors and interventions could be used to increase survival outcomes for RVF cases. The ARREST trial demonstrated that the use of extracorporeal membrane oxygenation (ECMO) was associated with improved outcomes compared to standard advanced care treatments (43% vs. 7%) in RVF patients.^9^ Moreover, findings from the ALPS trial highlighted that early administration of amiodarone compared to a placebo significantly increased the likelihood of survival to hospital discharge (37.1% vs. 28.0%) and survival with good functional recovery (31.6% vs. 23.3%) for RVF patients.^16^ The DOSE-VF trial also showed that both vector change and the use of double sequential defibrillation in RVF patients improved survival compared to standard defibrillation attempts.^17^ Although many regions are yet to adopt these interventions for RVF patients, amiodarone is already administered for OHCA in our region, and our observations correlate with those from the ALPS trial.^18^ Also, a trial involving prehospital ECMO CPR is currently underway in Victoria,^19^ contributing to the ongoing efforts to enhance the management and outcomes of RVF.

The definition used in our study reflects those used elsewhere,^6^, ^7^ and requires RVF patients to have three or more consecutive shocks which may be associated with longer resuscitation time. In our study, patients with RVF had a longer median resuscitation time compared to the non-RVF group. In OHCA, resuscitation time that exceeds 10 minutes is associated with poor outcomes, and survival is only 1% if resuscitation time exceeds 35 minutes.^20^, ^21^ To account for resuscitation time bias, we performed a sensitivity analysis which included resuscitation time in the multivariable models. Although increasing resuscitation time was associated with reduced odds of survival, it only moderately attenuated the impact of RVF on survival. This finding suggests that the impact of RVF on survival is independent of resuscitation time.

Community programs targeting public awareness of the use of public access defibrillators and delivering high-quality CPR are needed. Future research should focus on exploring factors that influence the rate of successful defibrillation on RVF patients including the optimal amount of energy used,^22^ pad position to lower transthoracic impedance,^23^ the influence of patient’s weight on shock success^22^ and shock impedance.^24^ There is also value in examining RVF in greater detail. It is not clear how patient characteristics and outcomes change with an increasing number of consecutive shocks. To date, existing studies have explored the relationship between total shocks and outcome,^25^, ^26^ and it is not known how representative these populations are of RVF cases.

### Limitations

Our study has several limitations. The study is retrospective in nature. The definition of RVF in our study may not be universally applicable, and it is possible that this definition included recurrent VF cases which could impact the accuracy of our findings. Additionally, approximately 2.5% of cases were missing RVF status and these cases were excluded from the analysis. Finally, treatments provided in hospitals are not reported which may influence patient outcomes.

## Conclusion

RVF accounts for 43% of initially shockable OHCA, although the incidence of RVF is declining. RVF is associated with a 50% reduction in the risk-adjusted odds of survival, however may be preventable with the early provision of bystander CPR and defibrillation. Community initiatives that focus on raising public awareness about the importance of administering CPR and the utilisation of publicly accessible defibrillators before EMS arrival are warranted. More research is also needed to examine the association between defibrillation characteristics and the likelihood of developing RVF.

## Sources of Funding

EN is supported by a 10.13039/501100000925National Health and Medical Research Council Postgraduate Scholarship (#2003449). ZN is supported by a Future Leader Fellowship from the National Heart Foundation of Australia (#105690).

## CRediT authorship contribution statement

**Abdulrahman Alhenaki:** Writing – original draft, Methodology, Formal analysis, Data curation, Conceptualization. **Zainab Alqudah:** Writing – review & editing, Supervision, Methodology, Formal analysis, Conceptualization. **Brett Williams:** Writing – review & editing, Supervision. **Emily Nehme:** Writing – review & editing, Data curation. **Ziad Nehme:** Writing – review & editing, Supervision, Methodology, Formal analysis, Conceptualization.

## Declaration of competing interest

The authors declare that they have no known competing financial interests or personal relationships that could have appeared to influence the work reported in this paper.
